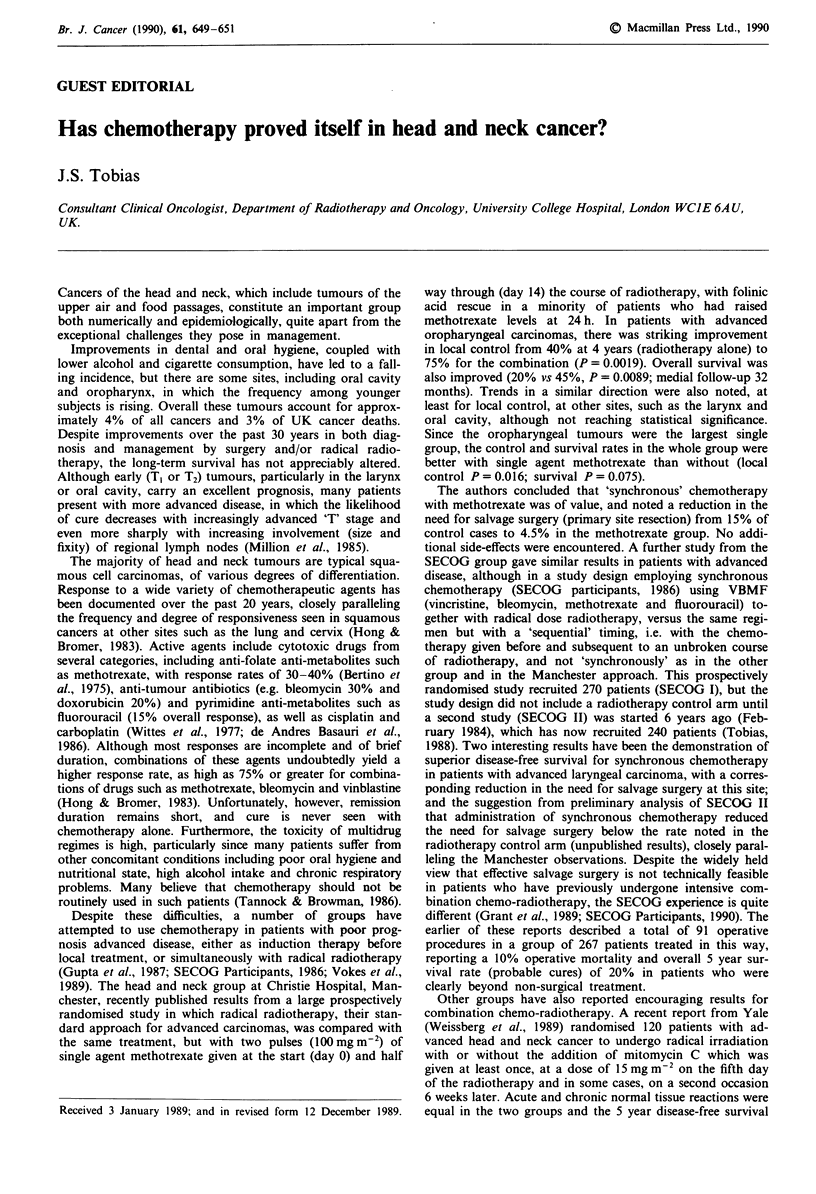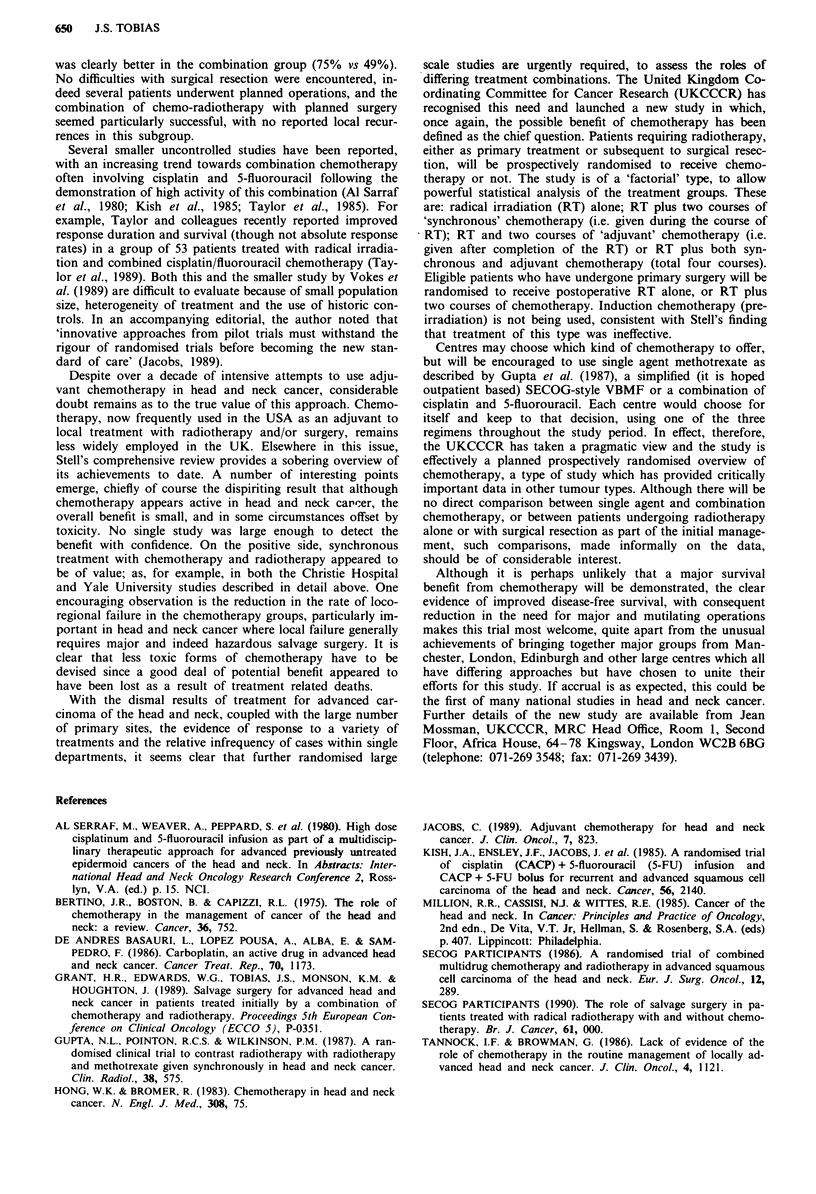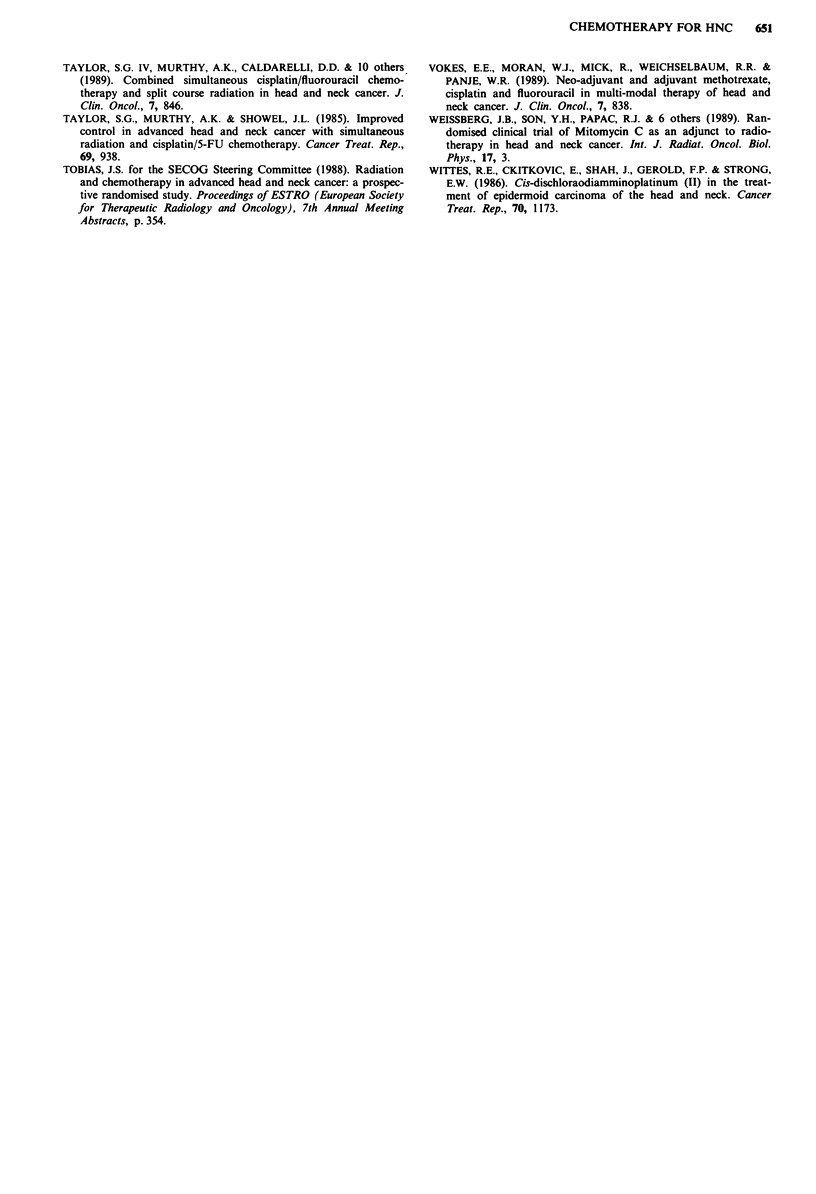# Has chemotherapy proved itself in head and neck cancer?

**DOI:** 10.1038/bjc.1990.146

**Published:** 1990-05

**Authors:** J. S. Tobias

**Affiliations:** Department of Radiotherapy and Oncology, University College Hospital, London, UK.


Br. J. Cancer (1990), 61, 649–651 © Macmillan Press Ltd., 1990

GUEST EDITORIAL

### Has chemotherapy proved itself in head and neck cancer?

J.S. Tobias

*Consultant Clinical Oncologist, Department of Radiotherapy and Oncology, University College Hospital, London WC1E 6AU, UK.*

Cancers of the head and neck, which include tumours of the upper air and food passages, constitute an important group both numerically and epidemiologically, quite apart from the exceptional challenges they pose in management.

Improvements in dental and oral hygiene, coupled with lower alcohol and cigarette consumption, have led to a falling incidence, but there are some sites, including oral cavity and oropharynx, in which the frequency among younger subjects is rising. Overall these tumours account for approximately 4% of all cancers and 3% of UK cancer deaths. Despite improvements over the past 30 years in both diagnosis and management by surgery and/or radical radiotherapy, the long-term survival has not appreciably altered. Although early ($T_1$ or $T_2$) tumours, particularly in the larynx or oral cavity, carry an excellent prognosis, many patients present with more advanced disease, in which the likelihood of cure decreases with increasingly advanced 'T' stage and even more sharply with increasing involvement (size and fixity) of regional lymph nodes (Million *et al.*, 1985).

The majority of head and neck tumours are typical squamous cell carcinomas, of various degrees of differentiation. Response to a wide variety of chemotherapeutic agents has been documented over the past 20 years, closely paralleling the frequency and degree of responsiveness seen in squamous cancers at other sites such as the lung and cervix (Hong & Bromer, 1983). Active agents include cytotoxic drugs from several categories, including anti-folate anti-metabolites such as methotrexate, with response rates of 30–40% (Bertino *et al.*, 1975), anti-tumour antibiotics (e.g. bleomycin 30% and doxorubicin 20%) and pyrimidine anti-metabolites such as fluorouracil (15% overall response), as well as cisplatin and carboplatin (Wittes *et al.*, 1977; de Andres Basauri *et al.*, 1986). Although most responses are incomplete and of brief duration, combinations of these agents undoubtedly yield a higher response rate, as high as 75% or greater for combinations of drugs such as methotrexate, bleomycin and vinblastine (Hong & Bromer, 1983). Unfortunately, however, remission duration remains short, and cure is never seen with chemotherapy alone. Furthermore, the toxicity of multidrug regimes is high, particularly since many patients suffer from other concomitant conditions including poor oral hygiene and nutritional state, high alcohol intake and chronic respiratory problems. Many believe that chemotherapy should not be routinely used in such patients (Tannock & Browman, 1986).

Despite these difficulties, a number of groups have attempted to use chemotherapy in patients with poor prognosis advanced disease, either as induction therapy before local treatment, or simultaneously with radical radiotherapy (Gupta *et al.*, 1987; SECOG Participants, 1986; Vokes *et al.*, 1989). The head and neck group at Christie Hospital, Manchester, recently published results from a large prospectively randomised study in which radical radiotherapy, their standard approach for advanced carcinomas, was compared with the same treatment, but with two pulses ($100\,mg\,m^{-2}$) of single agent methotrexate given at the start (day 0) and half way through (day 14) the course of radiotherapy, with folinic acid rescue in a minority of patients who had raised methotrexate levels at 24 h. In patients with advanced oropharyngeal carcinomas, there was striking improvement in local control from 40% at 4 years (radiotherapy alone) to 75% for the combination ($P = 0.0019$). Overall survival was also improved (20% *vs* 45%, $P = 0.0089$; medial follow-up 32 months). Trends in a similar direction were also noted, at least for local control, at other sites, such as the larynx and oral cavity, although not reaching statistical significance. Since the oropharyngeal tumours were the largest single group, the control and survival rates in the whole group were better with single agent methotrexate than without (local control $P = 0.016$; survival $P = 0.075$).

The authors concluded that 'synchronous' chemotherapy with methotrexate was of value, and noted a reduction in the need for salvage surgery (primary site resection) from 15% of control cases to 4.5% in the methotrexate group. No additional side-effects were encountered. A further study from the SECOG group gave similar results in patients with advanced disease, although in a study design employing synchronous chemotherapy (SECOG participants, 1986) using VBMF (vincristine, bleomycin, methotrexate and fluorouracil) together with radical dose radiotherapy, versus the same regimen but with a 'sequential' timing, i.e. with the chemotherapy given before and subsequent to an unbroken course of radiotherapy, and not 'synchronously' as in the other group and in the Manchester approach. This prospectively randomised study recruited 270 patients (SECOG I), but the study design did not include a radiotherapy control arm until a second study (SECOG II) was started 6 years ago (February 1984), which has now recruited 240 patients (Tobias, 1988). Two interesting results have been the demonstration of superior disease-free survival for synchronous chemotherapy in patients with advanced laryngeal carcinoma, with a corresponding reduction in the need for salvage surgery at this site; and the suggestion from preliminary analysis of SECOG II that administration of synchronous chemotherapy reduced the need for salvage surgery below the rate noted in the radiotherapy control arm (unpublished results), closely paralleling the Manchester observations. Despite the widely held view that effective salvage surgery is not technically feasible in patients who have previously undergone intensive combination chemo-radiotherapy, the SECOG experience is quite different (Grant *et al.*, 1989; SECOG Participants, 1990). The earlier of these reports described a total of 91 operative procedures in a group of 267 patients treated in this way, reporting a 10% operative mortality and overall 5 year survival rate (probable cures) of 20% in patients who were clearly beyond non-surgical treatment.

Other groups have also reported encouraging results for combination chemo-radiotherapy. A recent report from Yale (Weissberg *et al.*, 1989) randomised 120 patients with advanced head and neck cancer to undergo radical irradiation with or without the addition of mitomycin C which was given at least once, at a dose of $15\,mg\,m^{-2}$ on the fifth day of the radiotherapy and in some cases, on a second occasion 6 weeks later. Acute and chronic normal tissue reactions were equal in the two groups and the 5 year disease-free survival

Received 3 January 1989; and in revised form 12 December 1989.

was clearly better in the combination group (75% *vs* 49%). No difficulties with surgical resection were encountered, indeed several patients underwent planned operations, and the combination of chemo-radiotherapy with planned surgery seemed particularly successful, with no reported local recurrences in this subgroup.

Several smaller uncontrolled studies have been reported, with an increasing trend towards combination chemotherapy often involving cisplatin and 5-fluorouracil following the demonstration of high activity of this combination (Al Sarraf *et al*., 1980; Kish *et al*., 1985; Taylor *et al*., 1985). For example, Taylor and colleagues recently reported improved response duration and survival (though not absolute response rates) in a group of 53 patients treated with radical irradiation and combined cisplatin/fluorouracil chemotherapy (Taylor *et al*., 1989). Both this and the smaller study by Vokes *et al*. (1989) are difficult to evaluate because of small population size, heterogeneity of treatment and the use of historic controls. In an accompanying editorial, the author noted that 'innovative approaches from pilot trials must withstand the rigour of randomised trials before becoming the new standard of care' (Jacobs, 1989).

Despite over a decade of intensive attempts to use adjuvant chemotherapy in head and neck cancer, considerable doubt remains as to the true value of this approach. Chemotherapy, now frequently used in the USA as an adjuvant to local treatment with radiotherapy and/or surgery, remains less widely employed in the UK. Elsewhere in this issue, Stell's comprehensive review provides a sobering overview of its achievements to date. A number of interesting points emerge, chiefly of course the dispiriting result that although chemotherapy appears active in head and neck cancer, the overall benefit is small, and in some circumstances offset by toxicity. No single study was large enough to detect the benefit with confidence. On the positive side, synchronous treatment with chemotherapy and radiotherapy appeared to be of value; as, for example, in both the Christie Hospital and Yale University studies described in detail above. One encouraging observation is the reduction in the rate of loco-regional failure in the chemotherapy groups, particularly important in head and neck cancer where local failure generally requires major and indeed hazardous salvage surgery. It is clear that less toxic forms of chemotherapy have to be devised since a good deal of potential benefit appeared to have been lost as a result of treatment related deaths.

With the dismal results of treatment for advanced carcinoma of the head and neck, coupled with the large number of primary sites, the evidence of response to a variety of treatments and the relative infrequency of cases within single departments, it seems clear that further randomised large scale studies are urgently required, to assess the roles of differing treatment combinations. The United Kingdom Co-ordinating Committee for Cancer Research (UKCCCR) has recognised this need and launched a new study in which, once again, the possible benefit of chemotherapy has been defined as the chief question. Patients requiring radiotherapy, either as primary treatment or subsequent to surgical resection, will be prospectively randomised to receive chemotherapy or not. The study is of a 'factorial' type, to allow powerful statistical analysis of the treatment groups. These are: radical irradiation (RT) alone; RT plus two courses of 'synchronous' chemotherapy (i.e. given during the course of RT); RT and two courses of 'adjuvant' chemotherapy (i.e. given after completion of the RT) or RT plus both synchronous and adjuvant chemotherapy (total four courses). Eligible patients who have undergone primary surgery will be randomised to receive postoperative RT alone, or RT plus two courses of chemotherapy. Induction chemotherapy (pre-irradiation) is not being used, consistent with Stell's finding that treatment of this type was ineffective.

Centres may choose which kind of chemotherapy to offer, but will be encouraged to use single agent methotrexate as described by Gupta *et al*. (1987), a simplified (it is hoped outpatient based) SECOG-style VBMF or a combination of cisplatin and 5-fluorouracil. Each centre would choose for itself and keep to that decision, using one of the three regimens throughout the study period. In effect, therefore, the UKCCCR has taken a pragmatic view and the study is effectively a planned prospectively randomised overview of chemotherapy, a type of study which has provided critically important data in other tumour types. Although there will be no direct comparison between single agent and combination chemotherapy, or between patients undergoing radiotherapy alone or with surgical resection as part of the initial management, such comparisons, made informally on the data, should be of considerable interest.

Although it is perhaps unlikely that a major survival benefit from chemotherapy will be demonstrated, the clear evidence of improved disease-free survival, with consequent reduction in the need for major and mutilating operations makes this trial most welcome, quite apart from the unusual achievements of bringing together major groups from Manchester, London, Edinburgh and other large centres which all have differing approaches but have chosen to unite their efforts for this study. If accrual is as expected, this could be the first of many national studies in head and neck cancer. Further details of the new study are available from Jean Mossman, UKCCCR, MRC Head Office, Room 1, Second Floor, Africa House, 64–78 Kingsway, London WC2B 6BG (telephone: 071-269 3548; fax: 071-269 3439).

### References

AL SERRAF, M., WEAVER, A., PEPPARD, S. *et al*. (1980). High dose cisplatinum and 5-fluorouracil infusion as part of a multidisciplinary therapeutic approach for advanced previously untreated epidermoid cancers of the head and neck. In *Abstracts: International Head and Neck Oncology Research Conference 2*, Rosslyn, V.A. (ed.) p. 15. NCI.

BERTINO, J.R., BOSTON, B. & CAPIZZI, R.L. (1975). The role of chemotherapy in the management of cancer of the head and neck: a review. *Cancer*, **36**, 752.

DE ANDRES BASAURI, L., LOPEZ POUSA, A., ALBA, E. & SAMPEDRO, F. (1986). Carboplatin, an active drug in advanced head and neck cancer. *Cancer Treat. Rep*., **70**, 1173.

GRANT, H.R., EDWARDS, W.G., TOBIAS, J.S., MONSON, K.M. & HOUGHTON, J. (1989). Salvage surgery for advanced head and neck cancer in patients treated initially by a combination of chemotherapy and radiotherapy. *Proceedings 5th European Conference on Clinical Oncology (ECCO 5)*, P-0351.

GUPTA, N.L., POINTON, R.C.S. & WILKINSON, P.M. (1987). A randomised clinical trial to contrast radiotherapy with radiotherapy and methotrexate given synchronously in head and neck cancer. *Clin. Radiol*., **38**, 575.

HONG, W.K. & BROMER, R. (1983). Chemotherapy in head and neck cancer. *N. Engl. J. Med*., **308**, 75.

JACOBS, C. (1989). Adjuvant chemotherapy for head and neck cancer. *J. Clin. Oncol*., **7**, 823.

KISH, J.A., ENSLEY, J.F., JACOBS, J. *et al*. (1985). A randomised trial of cisplatin (CACP) + 5-fluorouracil (5-FU) infusion and CACP + 5-FU bolus for recurrent and advanced squamous cell carcinoma of the head and neck. *Cancer*, **56**, 2140.

MILLION, R.R., CASSISI, N.J. & WITTES, R.E. (1985). Cancer of the head and neck. In *Cancer: Principles and Practice of Oncology*, 2nd edn., De Vita, V.T. Jr, Hellman, S. & Rosenberg, S.A. (eds) p. 407. Lippincott: Philadelphia.

SECOG PARTICIPANTS (1986). A randomised trial of combined multidrug chemotherapy and radiotherapy in advanced squamous cell carcinoma of the head and neck. *Eur. J. Surg. Oncol*., **12**, 289.

SECOG PARTICIPANTS (1990). The role of salvage surgery in patients treated with radical radiotherapy with and without chemotherapy. *Br. J. Cancer*, **61**, 000.

TANNOCK, I.F. & BROWMAN, G. (1986). Lack of evidence of the role of chemotherapy in the routine management of locally advanced head and neck cancer. *J. Clin. Oncol*., **4**, 1121.

TAYLOR, S.G. IV, MURTHY, A.K., CALDARELLI, D.D. & 10 others (1989). Combined simultaneous cisplatin/fluorouracil chemotherapy and split course radiation in head and neck cancer. *J. Clin. Oncol.*, **7**, 846.

TAYLOR, S.G., MURTHY, A.K. & SHOWEL, J.L. (1985). Improved control in advanced head and neck cancer with simultaneous radiation and cisplatin/5-FU chemotherapy. *Cancer Treat. Rep.*, **69**, 938.

TOBIAS, J.S. for the SECOG Steering Committee (1988). Radiation and chemotherapy in advanced head and neck cancer: a prospective randomised study. *Proceedings of ESTRO (European Society for Therapeutic Radiology and Oncology), 7th Annual Meeting Abstracts*, p. 354.

VOKES, E.E., MORAN, W.J., MICK, R., WEICHSELBAUM, R.R. & PANJE, W.R. (1989). Neo-adjuvant and adjuvant methotrexate, cisplatin and fluorouracil in multi-modal therapy of head and neck cancer. *J. Clin. Oncol.*, **7**, 838.

WEISSBERG, J.B., SON, Y.H., PAPAC, R.J. & 6 others (1989). Randomised clinical trial of Mitomycin C as an adjunct to radiotherapy in head and neck cancer. *Int. J. Radiat. Oncol. Biol. Phys.*, **17**, 3.

WITTES, R.E., CKITKOVIC, E., SHAH, J., GEROLD, F.P. & STRONG, E.W. (1986). *Cis*-dischloraodiamminoplatinum (II) in the treatment of epidermoid carcinoma of the head and neck. *Cancer Treat. Rep.*, **70**, 1173.